# Overcoming therapeutic resistance to platinum-based drugs by targeting Epithelial–Mesenchymal transition

**DOI:** 10.3389/fonc.2022.1008027

**Published:** 2022-10-14

**Authors:** Xirui Duan, Maochao Luo, Jian Li, Zhisen Shen, Ke Xie

**Affiliations:** ^1^ Department of Oncology, Sichuan Academy of Medical Sciences and Sichuan Provincial People’s Hospital, School of Medicine, University of Electronic Science and Technology of China, Chengdu, China; ^2^ State Key Laboratory of Biotherapy and Cancer Center, West China Hospital, and Collaborative Innovation Center for Biotherapy, Chengdu, China; ^3^ West China School of Basic Medical Sciences and Forensic Medicine, Sichuan University, Chengdu, China; ^4^ Department of Otorhinolaryngology and Head and Neck Surgery, The Affiliated Lihuili Hospital, Ningbo University, Ningbo, China

**Keywords:** platinum-based drugs, epithelial-mesenchymal transition, drug resistance, targeted therapy, cancer stem cells

## Abstract

Platinum-based drugs (PBDs), including cisplatin, carboplatin, and oxaliplatin, have been widely used in clinical practice as mainstay treatments for various types of cancer. Although there is firm evidence of notable achievements with PBDs in the management of cancers, the acquisition of resistance to these agents is still a major challenge to efforts at cure. The introduction of the epithelial-mesenchymal transition (EMT) concept, a critical process during embryonic morphogenesis and carcinoma progression, has offered a mechanistic explanation for the phenotypic switch of cancer cells upon PBD exposure. Accumulating evidence has suggested that carcinoma cells can enter a resistant state *via* induction of the EMT. In this review, we discussed the underlying mechanism of PBD-induced EMT and the current understanding of its role in cancer drug resistance, with emphasis on how this novel knowledge can be exploited to overcome PBD resistance *via* EMT-targeted compounds, especially those under clinical trials.

## Introduction

Metallodrugs play essential roles in anticancer therapy, where platinum-based drugs (PBDs) are most widely used. More than 40 years ago, the United States Food and Drug Administration (FDA) approved the platinum (Pt) compound, cisplatin, to treat ovarian cancer, bladder cancer, and metastatic testicular cancer (1). With the wide application of PBDs, these drugs have become the first-line agents in anticancer therapy for other types of malignancies (2). Despite the prospective effect, resistance to PBDs has become the main reason for chemotherapy failure in clinical treatments.

**Figure 1:**
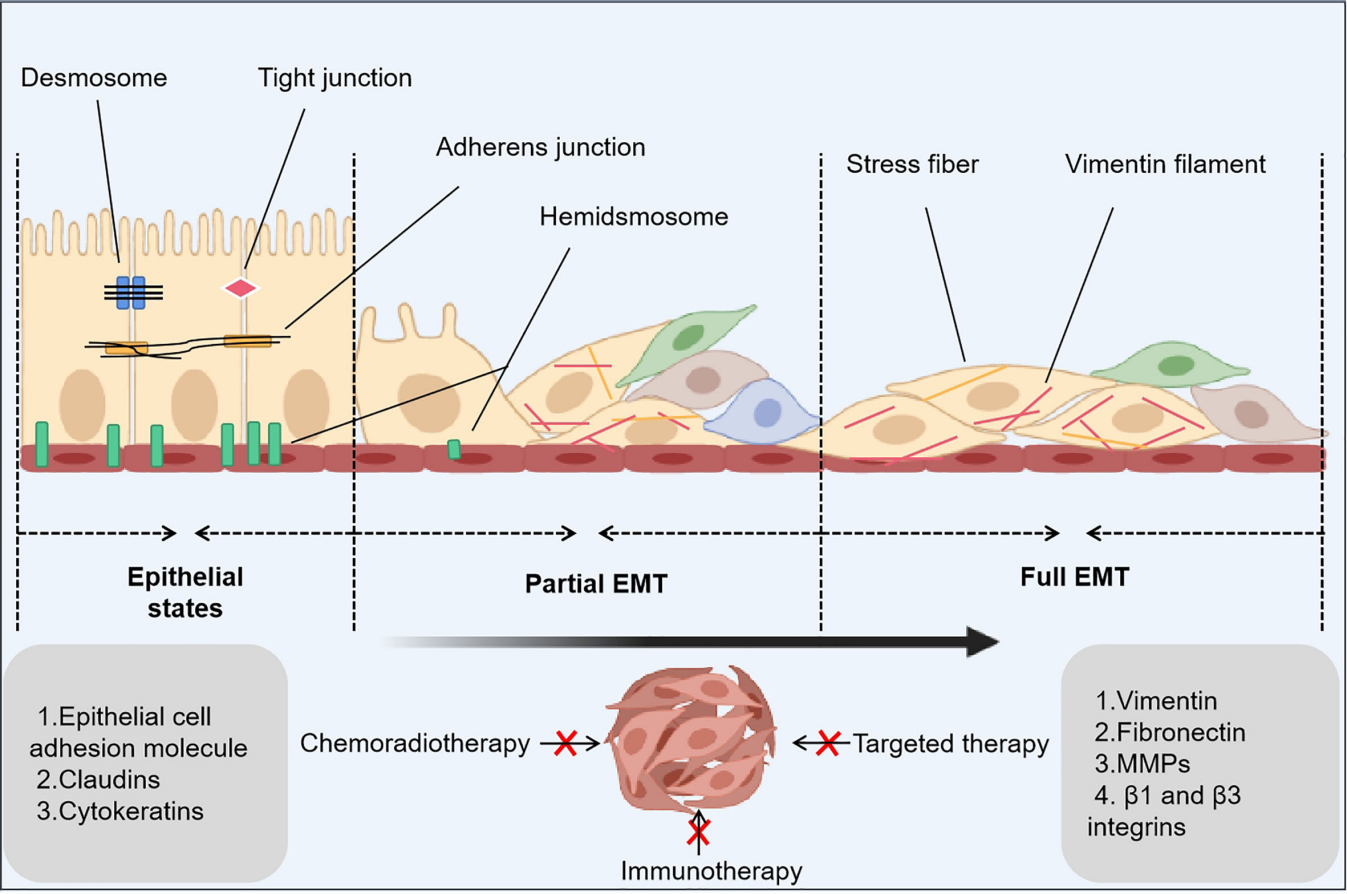
EMT-related principles underlying cancer drug resistance. The epithelial-mesenchymal transition (EMT) is a dynamic process that consists of three states: an epithelial state with epithelial phenotypes, a hybrid state with epithelial phenotypes and mesenchymal phenotypes, and a mesenchymal state with mesenchymal phenotypes. In the latter two states, a small number of cancer stem cells and drug-tolerant persister cells occur to inhibit anticancer treatments.

Numerous studies have demonstrated that the epithelial-mesenchymal transition (EMT) is a primary cause of PBD resistance in cancer cells. The EMT is a process in which cells lose the epithelial marker E-cadherin and express the mesenchymal marker vimentin. It is involved in embryogenesis, tissue morphogenesis, gastrulation, and wound repair (3). Multiple studies have revealed that cancerous tissue has both epithelial and mesenchymal characteristics (4). During the EMT process, the connection between cells is weakened, cell motility is enhanced, and the synthesis of multidrug-resistant proteins is increased, which makes the tumor tissue show the characteristics of high invasiveness and metastasis and high drug resistance. Cancer stem cells (CSCs), a minor population of cancer cells, are regulated by EMT, and these cells play an important role in invasion, metastasis, drug resistance, and phenotypic cell switching (5). With the current deeper exploration of oncology and cancer-related problems, researchers have found that the EMT is closely related to drug resistance, tumor stemness and aggressiveness, and metastasis (**Figure 1**) (6).

Development of the EMT phenotype is one of the fundamental reasons that tumor cells transform from complete sensitivity to extreme resistance during PBD chemotherapy. In this review, we attempt to synthetically illustrate the underlying mechanism of EMT-induced resistance to metallodrugs. In addition, we highlight novel, potentially effective strategies that combine targeted molecular inhibitors to obstruct the EMT or inhibit EMT-related cellular changes to overcome drug resistance and improve the antitumor effects of Pt compounds (**Table 1** and **2**).

**Table 1:** Classes of synthetic drugs targeting epithelial-mesenchymal transition.

Drug	Mechanism of action	Clinical trial for	Ref
Thymoquinone	β-catenin inhibitor	Bladder cancer	(7)
Luteolin	Notch-1 inhibitor	Gastric cancer	(8)
	β-catenin inhibitor	Breast cancer	
Curcumin	Snail inhibitor	Oral cancer	(9)
	CXCR4 inhibitor	Colorectal cancer	
	Hedgehog inhibitor	Pancreatic cancer	
	TGF-β inhibitor	Hepatoma	
		retinal pigment epithelial cancer	
	ERK inhibitor	Hepatocellular carcinoma	
	NF-κB inhibitor	Breast cancer	
	STAT3 inhibitor	Melanoma	
	ERK1/2 inhibitor	Bladder cancer	
	JNK inhibitor		
	MAPK inhibitor		
	Smad2/3 inhibitor	Thyroid cancer	
Glucosamine	NF-κB inhibitor	Breast cancer	(10)
Ginsenosides	NF-κB inhibitor	Breast cancer	(11)
Combretastatin	Akt inhibitor	Thyroid papillary carcinoma	(12)
Oxyresveratrol	Snail inhibitor	Colon cancer	(13)
N‐arachidonoyl dopamine	ERK1/2 inhibitor	Breast cancer	(14)
Kaempferol	Snail inhibitor	NSCLC	(15)
		Breast cancer	
Doxycycline	Snail1 inhibitor	Breast cancer	(16)
	twist1 inhibitor		
	ZEB1 inhibitor		
	Slug inhibitor		
Resveratrol	Hedgehog inhibitor	Gastric Cancer	(17)
Genistein	Twist1 inhibitor		
Pantoprazole	β-catenin inhibitor		
Astragaloside	TGF-β1 inhibitor		
Kaempferol	Akt inhibitor	Cervical cancer	(18)
Niclosamide	β-catenin inhibitor	Colon cancer	(19)
Lipoic acid	TGF-β inhibitor	Breast cancer	(20)
Piperlongumine	Snail1 inhibitor	Epithelial cancer	(21)
	Twist1 inhibitor		
Manganese-12 acetate	β-catenin inhibitor	Breast cancer	(22)
	Akt inhibitor		
Shikonin	Akt inhibitor	Glioblastoma	(23)
Actinomycin V	Snail inhibitor	Breast cancer	(24)
	Slug inhibitor		
Hydroxytyrosol	β-catenin inhibitor	Breast cancer	(25)
	Smad2/3 inhibitor		
Antrodia camphorata	Akt inhibitor	Ovarian cancer	(26)
	β-catenin inhibitor	Colon cancer	
Quercetin	β-catenin inhibitor	Colon cancer	(27)

**Table 2:** Classes of natural drugs targeting epithelial-mesenchymal transition.

Drug	Mechanism of action	Clinical trial for	Ref
Sulforaphane	ERK5 activator	Lung cancer	(28)
Nitidine chloride	JAK2/STAT3 inhibitor	Glioblastoma	(29)
Eribulin	TGF-β/Smad2/3 inhibitor	Triple-negative breast cancer	(30)
Ursolic acid	TGF-β1/smad3 inhibitor	Renal fibrosis	(31)
Curcumin	TGF-β/Smad inhibitor	Hepatic fibrosis	(32)
Celastrol	TGF-β1/smad2/3 inhibitor	Pulmonary Fibrosis	(33)
Baicalin	ERK inhibitor	Hepatocellular Carcinoma	(34)
Isoviolanthin	TGF-β/Smad inhibitor	Hepatocellular Carcinoma	(35)
	PI3K/Akt/mTOR inhibitor		
Astragaloside IV	Akt/GSK-3β/β-catenin inhibitor	Hepatocellular Carcinoma	(36)

## Mechanisms involved in PBD-induced EMT

**Figure 2:**
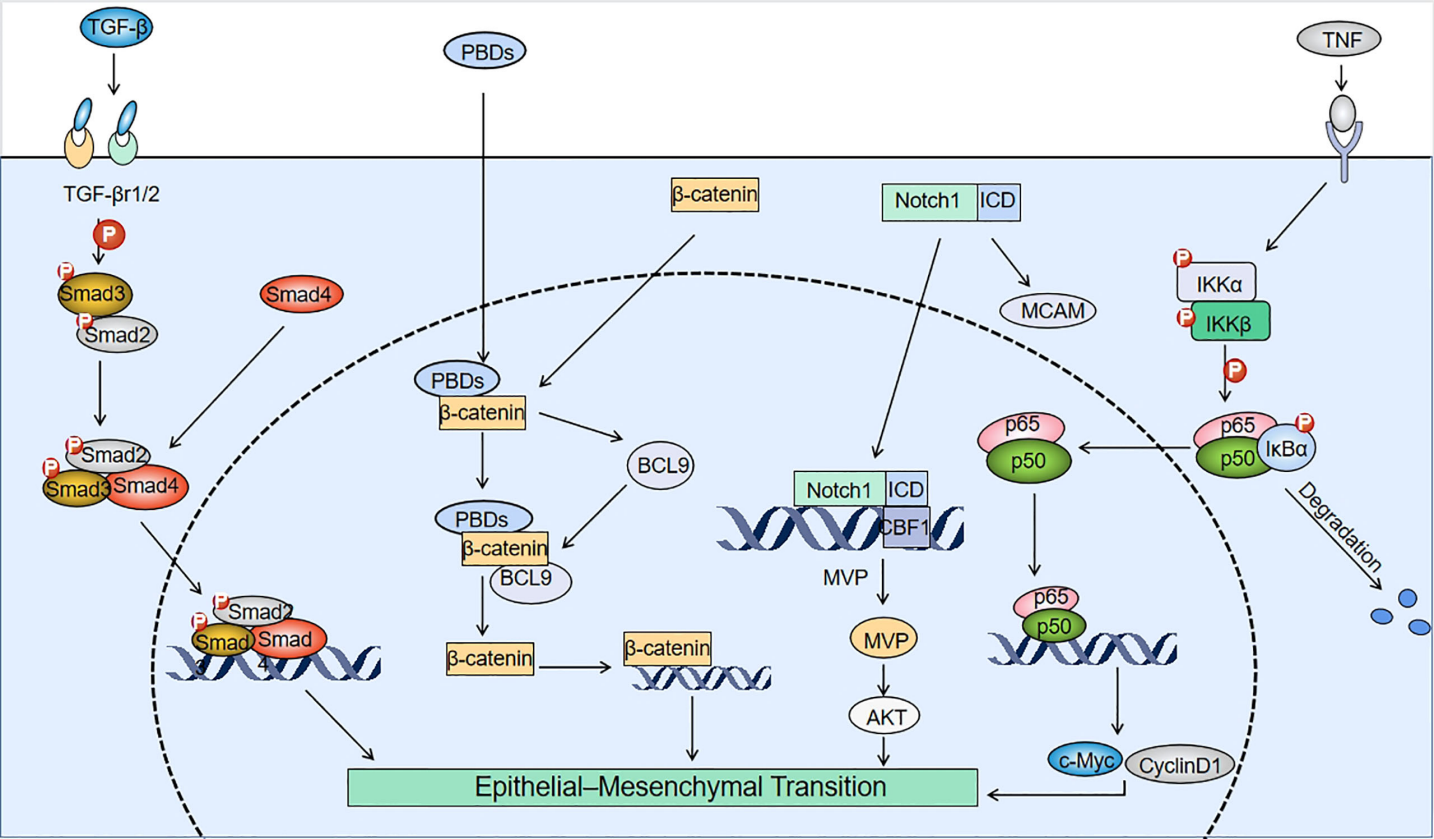
Mechanisms involved in PBD-induced EMT. TGF-β interacts with TGF-β receptors (TGF-βr1/2) to induce phosphorylation of Smad2/3, which binds to Smad4 to form a Smad2/3/4 complex. After platinum-based drugs (PBDs) enter tumor cells, they bind to the N-terminus of β-catenin and upregulate BCL9. BCL9 can form a complex with β-catenin to create positive feedback that promotes β-catenin expression, and induces the epithelial-mesenchymal transition (EMT). The pathways for Notch1 to induce EMT include: promoting melanoma cell adhesion molecule (MCAM) expression and binding of the Notch1 internal domain (ICD) to the CBF1 site of the major vault protein (*MVP*) gene to upregulate MVP. The binding of tumor necrosis factor (TNF) to membrane surface receptors drives IkB kinase (IKK) to phosphorylate IkB. Degradation of IkB forms the p65/p50 dimeric complex that binds to EMT-related genes.

When cancer cells receive signals from tumor-associated reactive stroma (e.g., Wnt, TGF-β, and Notch signaling), the expression of EMT-inducible transcription factors is elevated and this can activate the EMT (37). When treating cancer patients with chemotherapeutic drugs, some cancer cells develop drug resistance, and the resistance of cancer cells is negatively correlated with patient survival. Moreover, recent findings have shown that drug-resistant tumor cells tend to undergo EMT (38). This section summarizes developments in the last five years regarding the signaling pathways associated with the induction of EMT by PBDs (**Figure 2**). Other scholars have demonstrated the effects of mitotic kinases such as Nek2 and Mps1 (TTK) on EMT, focusing on AuroraA, AuroraB, Bub1 and Hec1 (high expression in cancer) as potential targets for cancer therapeutic intervention through their impact on EMT. The established relationships and interactions between these and other mitotic kinases are highlighted, together with the impact of classical signaling pathways and long RNAs on EMT. Recent studies have found that microbial metabolism can increase the intake of 5-fluorouracil (5FU) to counteract the drug resistance in CRC by modulating the FoxO3-FOXM1 axis to alter drug transporter sensitivity. The researchers also found that this process involved changes in the expression of EMT mediators (39). There have been few studies on the relationship between EMT and drug transporters, and regulating drug transporters (drug import/export pumps) is expected to become a new approach and research direction for anti-EMT treatment.

### Wnt

The Wnt signaling pathway is a complex regulatory network that includes the Wnt/β-catenin signaling pathway, the Wnt/PCP pathway (planner-cell polarity pathway), and the Wnt/Ca2+ pathway (40). PBDs induce EMT mainly by activating the classical Wnt signaling pathway. The expression of c-MYB, a proto-oncogene, is increased in drug-resistant cells, which directly induces EMT by promoting miR-21 expression to increase the mesenchymal marker zinc finger E-box binding homeobox 1 (ZEB1) and decrease E-cadherin expression. By activating the Wnt pathway, c-MYB can also inhibit β-catenin phosphorylation and thus indirectly induce EMT (41). It has been found that in non-small cell lung cancer (NSCLC), PBDs can increase intracellular B-cell CLL/lymphoma 9 (BCL9) expression and form the β-catenin/BCL9 complex by selectively binding to the N-terminal structural domain of β-catenin. This binding enhances the transcriptional activity of Wnt signaling and promotes the transcription of β-catenin, thus increasing nuclear translocation and inducing EMT (42). It has also been shown that overexpression of BMAL1 (the central positive loop element that initiates circadian oscillations) and the PDZ-binding motif (TAZ) can induce the EMT by promoting nuclear expression of β-catenin in chemo-resistant colorectal cancer (CRC) cells (38, 43). Ectopic expression of disheveled (DVL) was effective in activating the Wnt/β-catenin signaling pathway even in the absence of Wnt ligand stimulation. Indeed, the forkhead box M1 (FOXM1) induction of Snail expression, metastasis, and chemoresistance requires DVL2, whereas FOXM1 does not alter DVL2 expression (44).

In addition, miR-28-5p expression was also decreased in drug-resistant CRC cells, thereby attenuating the inhibitory effect on downstream structure-specific recognition protein-1 (SSRP1) and increasing its expression to promote EMT by activating the Wnt/β-catenin pathway (45). The expression of miR-302 was reduced in drug-resistant cells, which directly targeted and upregulated ATPase family AAA domain containing 2 (ATAD2). This alteration significantly downregulated the tumor suppressor APC and upregulated nuclear-catenin *via* activation of the Wnt/β-catenin signaling pathway. The result was a decrease in expression of E-cadherin and increase in expression of vimentin (46). In drug-resistant NSCLC cells, miR-192 and miR-662 were overexpressed. MiR-192 may activate the Wnt pathway by silencing its atypical inhibitors (e.g., NOTUM) and upregulating FOXM1 expression. In parallel, miR-662 may enhance Wnt by upregulating the forkhead box N3 (FOXN3), forkhead box protein 1 (FOXP1), and receptor tyrosine kinase-like orphan receptor 1 (ROR1), and glioma-associated oncogene protein-2 (GLI2) signaling pathways and thus promote EMT. Similarly, miR-192 and miR-662 could upregulate the expression of the lncRNA, CASC9, and promote EMT in gastric cancer cells (47). In addition, BCL9 expression was also increased in drug-resistant NSCLC cells, activating the EMT mechanism by stimulating nuclear translocation of β-catenin (48).

### TGF-β

Transforming growth factor-β (TGF-β) is a multifunctional cytokine in mammals consisting of three isoforms, TGF-β1, TGF-β2, and TGF-β3. TGF-β binds to the TGF-β receptor type 1 complex (TGFβ-R1 and TGFβ-R2), leading to phosphorylation of the Smad2/3 protein in the cytoplasm. Smad2/3 interacts with Smad4 to form the Smad2/3/4 complex, which is translocated to the nucleus where it activates transcription of TGF-β-responsive downstream genes. This leads to activation of the signaling cascade that induces EMT (49). Cisplatin has been shown to increase IL-6 release and TGF-β expression in cancer-associated fibroblasts. IL-6 blocked apoptosis during inflammation, which could also protect cancer cells from apoptosis and chemotherapeutic agents. In addition, IL-6 enhanced the TGF-β-induced EMT in NSCLC. MiRNA-17, 20a, and 20b were expressed at low levels in cancer cells, and could also activate the TGF-β pathway (50). In the following section, we will describe the detailed mechanism of platinum-induced activation of the TGF-β signaling pathway.

Following cisplatin treatment of lung cancer cells, the C-X-C motif chemokine ligands/CXC chemokine receptor (CXCLs/CXCR2) signaling pathway was altered in resistant cells, resulting in increased expression of CXCR2-associated chemokines (CXCL1, CXCL2, and CXCL5), increased neutrophil infiltration and concomitant upregulation of the immunosuppressive factors, TGF-β and arginase (Arg-1). These factors induced neutrophil polarization toward the N2 type, which has a significantly reduced tumor-killing capacity. These cells then produced more TGF-β and Arg-1, suppressing the antitumor immune response, and thereby inducing EMT and promoting tumor progression (51). In drug-resistant SCLC cells, epithelial splicing regulatory protein 1 (ESRP1) expression was significantly downregulated, which increased the proportion of full-length CARM1 (CARM1FL) by regulating the selective splicing of coactivator associated arginine methyltransferase 1 (CARM1). This inhibited arginine methylation of Smad7, activated the TGF-β/Smad pathway, and increased Smad3 phosphorylation that promoted the EMT (52). Increased expression of CD24 in drug-resistant ovarian cancer cells led to enhanced TGF-β signaling. This TGF-β signaling cascade enabled the activation of downstream PI3k/Akt and MAPK signaling pathways, which further increased Snail expression and decreased E-cadherin expression, leading to the development of EMT (53). In patients with nasopharyngeal carcinoma who were treated with cisplatin, overexpression of miR-449b led to the degradation of TGF-β1 mRNA, resulting in a decrease in TGF-β1 expression. This led to downregulation of miR-34c, which directly induced SRY-box transcription factor 2 (SOX2) upregulation and promoted EMT (54). Bone morphogenetic protein 4 (BMP4), a member of the TGF-β superfamily, was overexpressed in drug-resistant hepatocellular carcinoma (HCC). This significantly reduced the expression of Bax proapoptotic protein and caspase-3 and significantly enhanced the expression of Bcl-2 antiapoptotic protein. BMP4 induced cyclin B1 and cyclin-dependent kinase 1 (CDK1) expression in HCC cells, thereby promoting cell cycle progression from G2 to M and resisting the oxaliplatin-derived G2/M blockade. BMP4 also promoted EMT by increasing the activity of matrix metalloproteinases (MMPs), upregulating vimentin expression, and downregulating E-cadherin (55). In drug-resistant oral squamous cell carcinoma (OSCC), circANKS1B was overexpressed and attenuated the direct inhibitory effect of miR-515-5p on TGF-1 by sequestering miR-515-5p, leading to activation of the TGF-β pathway. TGF-β pathway activation increased N-cadherin expression and decreased E-cadherin expression, resulting in the development of EMT (56). Notably, esophageal adenocarcinoma (EAC) cells were capable of producing and secreting large amounts of active TGF-β under the high therapeutic stress of chemotherapy, thereby inducing EMT. The reversal of EMT in EAC cells could not be achieved by short-term drug abstinence, suggesting that the drug dormancy period (i.e., intermittent radiotherapy) was insufficient to prevent radiotherapy-induced EMT. The addition of the TGF-neutralizing antibody fresolimumab during the second week of radiotherapy, however, was able to block the EMT in EAC cells and improve therapeutic efficacy (57).

### Notch

The NOTCH pathway has been highly conserved during evolution and is involved in controlling cell proliferation and inhibiting apoptosis (58). In mammals, the NOTCH pathway has four receptors (NOTCH1, 2, 3, and 4) and five ligands (JAG1 and 2, DLL1, 3, and 4), all of which are type 2 transmembrane proteins (59). Following receptor-ligand binding in the NOTCH pathway, the γ-secretase complex releases the intracellular structural domain of the NOTCH receptor, which translocates into the nucleus and induces expression of target genes, such as the HES/HEY family (60). A large body of evidence suggests that the NOTCH pathway is involved in the induction of EMT in normal and tumor tissues. In drug-resistant head and neck squamous cell carcinoma (HNSCC), increased NOTCH4 expression and resulted in specific upregulation of the downstream gene Hes-related family basic helix-loop-helix transcription factor with YRPW motif 1 (HEY1) expression, without affecting other NOTCH downstream genes. This resulted in reduced E-cadherin expression and increased expression of vimentin, fibronectin, Twist-related protein 1 (TWIST1), and SOX2, which induced EMT. HEY1 inhibition also inversely reduced NOTCH4 expression in HNSCC (60). In CR cells, TAZ overexpression increased the activation of the downstream signaling molecule Notch1 (38). In addition, Notch1 expression was increased in resistant cells after carboplatin treatment of triple-negative breast cancer (TNBC). On the one hand, Notch1 could promote melanoma cell adhesion molecule (MCAM) expression through direct activation of the MCAM promoter, thereby inducing EMT (61). On the other hand, the Notch1 intracellular structural domain (ICD) bound to the CBF1 binding site on the MVP (major vault protein) promoter, thereby upregulating the expression of MVP and activating the AKT pathway to promote EMT (62). In addition, in high-grade serous ovarian cancer, the Notch3 pathway was activated, with increased expression of downstream SUSD2. This, in turn, induced the EMT by reducing E-cadherin expression through increased epithelial cell adhesion molecule (EpCAM) expression (63). Low concentrations of cisplatin (DDP, CDDP) could induce EMT in osteosarcoma cells *via* the Notch signaling pathway by promoting the expression of NOTCH2 and its target gene HEY1 (64).

### NF-κB

NF-κB proteins commonly form homo/heterodimers with p65 and p50 and are inactivated in the cytoplasm by binding to the inhibitory protein IkB to form a trimeric complex (65). When the upstream signaling factor, tumor necrosis factor (TNF), binds to its cell membrane surface receptor, the receptor conformation changes and signals IKK (IkB kinase) kinase to phosphorylate IkB and dissociate it from the trimer. The NF-κB dimer then exposes the nuclear localization sequence (NLS) and rapidly moves from the cytoplasm into the nucleus, where it binds to specific sequences on the nuclear DNA and promotes the transcription of related genes, such as cyclinD1, c-Myc, matrix metalloproteinase-9 (MMP-9), and vascular endothelial growth factor (VEGF), which are EMT-related proteins (66). Studies have shown that aberrant activation of NF-κB could induce the development of EMT in drug-resistant cells. The expression of EGFR was increased in drug-resistant head and neck squamous cell carcinoma (HNSCC), resulting in increased IKKβ expression and activation of the downstream NF-κB signaling pathway. NF-κB increased downstream signal transducer and activator of transcription 3 (STAT3) by promoting IL-6 expression, which induced overexpression of N-cadherin and decreased E-cadherin expression, promoting EMT (67). In CRC cells, increased DJ-1 expression promoted EMT by activating the NF-κB/Snail signaling pathway and increasing Snail protein expression (48). In triple-negative breast cancer (TNBC) cells, cisplatin-induced activation of the ERK1/2-p90RSK signaling pathway led to NF-κB activation, which promoted the expression of Snail Twist and ZEB-1 and induced EMT (68).

### Hedgehog

The hedgehog signaling pathway is important for cellular self-renewal, tissue maintenance, and cell regeneration. The GLI family is a transcription factor for hedgehog signaling that includes glioma-associated oncogene homolog 1 (GLI1), GLI2, and GLI3. GLI1 usually acts as a potent target activator, while GLI2 and 3 have dual functions as repressors or activators depending on the situation (69). In cisplatin-resistant breast cancer cells, elevated expression of ubiquitin-specific peptidase 37 (USP37) upregulated the expression of purmorphamine (PM). Expression of the Hh targets (Smo and gli1) and the cell proliferation marker Ki-67 were also elevated through the hedgehog signaling pathway, resulting in significant upregulation of Snail1, N-cadherin, and vimentin, downregulation of E-cadherin, and induction of EMT (70). In oral squamous cell carcinoma (OSCC), drug-resistant cells enhanced CSC-associated features and increased CD10 expression, resulting in increased expression of Smo and gli1, which activated the hedgehog signaling pathway and induced EMT (71). In addition, cisplatin induced the expression of prostaglandin E2 (PGE2) in osteoclast cells, which promoted GLI1 expression by activating integrin β1. GLI1, in turn, promoted expression of B cell-specific Moloney murine leukemia virus integration site 1(BMI1) by activating the hedgehog signaling pathway, thereby altering the microenvironment and inducing CSC-like features that promoted EMT (69).

### PI3K/AKT/mTOR

PI3K (phosphatidylinositol kinase) is a dimer consisting of the regulatory subunit p85 and the catalytic subunit p110. When it binds to growth factor receptors (EGFR), it alters the protein structure of Akt and activates it, promoting or inhibiting the activity of a range of downstream substrates such as the apoptosis-associated proteins Bad and caspase9 by phosphorylation, thereby regulating cell proliferation, differentiation, apoptosis and migration phenotypes. In addition, Akt also activated IKK, which interacted with the NF-kB pathway. In this section, we describe the molecular changes that activate the PI3K/AKT signaling pathway in drug-resistant cells from different cancers.

After cisplatin treatment of non-small cell lung cancer (NSCLC), the expression of FOXC2 was increased, inactivating the pro-apoptotic factor GSK-3β by activating AKT. This leads to stabilization and nuclear localization of Snail, increasing the expression of the mesenchymal markers vimentin and N-cadherin, and decreasing the expression of the epithelial marker E-cadherin, thereby promoting EMT (48, 72). Increased expression of *FAM83D*, a mitosis-related gene, activated the AKT/mTOR signaling pathway, resulting in increased phosphorylation of p70s6k, downregulation of the epithelial markers E-cadherin and α-catenin, and upregulation of the mesenchymal markers N-cadherin and vimentin, thereby inducing EMT (73). Increased expression of PAX6 activated the downstream PI3K/AKT signaling pathway by directly binding to the promoter region of ZEB2 and upregulating its expression (74). Similarly, increased expression of the lncRNA, UCA1 induced EMT by activating the AKT/mTOR pathway (75).

In nasopharyngeal carcinoma cells, cisplatin treatment caused specific overexpression of miR-205-5p in drug-resistant cells, which promoted NPC cell proliferation and decreased PTEN (a classical tumor suppressor) expression by targeting it through phosphorylation of the PI3K/AKT signaling pathway. Low expression of PTEN induced the absence or reduction of epithelial markers (E-cadherin) and increased expression of mesenchymal markers (vimentin and N-cadherin) through the regulation of Snail/Slug, thereby promoting EMT and increasing the migration and invasive capacity of NPC cells. MiR-205-5p promoted cell metastasis by upregulating MMP-9 and MMP-2, which caused ECM degradation (76). Furthermore, increased expression of miR-BART22 increased MYH9 expression through activation of the PI3K/AKT/c-Jun axis. MYH9 then induced GSK-3β degradation and mediated nuclear translocation of β-catenin, thereby stimulating the EMT machinery (48). The PI3K/Akt pathway was activated in drug-resistant gastric cancer cells, leading to an increase in downstream Rac1 activity, which further activated the JNK pathway and promoted self-renewal of GA CSCs and EMT (EMT may be a mechanism for generating CSCs). At the same time, Rac1 led to increased expression of N-cadherin and Slug. In GA cells, inhibition of any component of the PI3K/Akt-Rac1/JNK axis could block expression of the EMT transcription factor, Slug, and inhibit EMT (77).

### Long non-coding RNAs

Long non-coding RNAs (lncRNAs) are defined as RNA transcripts longer than 200 nucleotides that do not encode polypeptides. Studies have shown that lncRNAs and miRNAs play a role in promoting the continuing proliferation of tumor cells and drug resistance (78); therefore, they are considered key regulators of cancer pathways and are widely studied as biomarkers of disease (79). One of the important aspects is the impact of miRNA and miRNA-lncRNA competition on tumor EMT progression.

MiR-574-3p has a tumor-suppressive effect on gastric cancer cells. In cisplatin-resistant cancer cells, miR-574-3p expression decreased and increased the expression of ZEB1 protein by attenuating the direct inhibition of ZEB1 protein, which in turn inhibited the transcription of E-cadherin, reduced its expression, increased the expression of vimentin, and induced EMT (80). In addition, miR-492 overexpression promoted CSCs by directly targeting the 3’ UTR of DNMT3B and inhibiting its expression, thereby inducing EMT (81). MiR-17 expression was also increased, which promoted EMT by downregulating DEDD (82). In ovarian cancer (OC) cells, decreased expression of miR-1294 allowed an increase in IGF1-R expression, which induced the EMT (48). The expression of miR-363 was also decreased, promoting EMT by attenuating the direct inhibitory effect on Snail (83, 84). In contrast, the expression of miR-149-3p was increased, promoting EMT by downregulating TIMP2 and CDKN1A expression (85). Notably, in OC cells, there is a miR-374b-5p-FOXP1 feedback loop in which miR-374b-5p can negatively regulate FOXP1 by binding to the 3’UTR of FOXP1, which can also repress miR-374b-5p transcription. In poor prognosis cells, the expression of miR-374b-5p was downregulated, while FOXP1 expression was upregulated, thereby inducing EMT (86). The competition between miRNA and lncRNA is also closely related to EMT. lncRNA-ATB is a ceRNA that is a regulator of the TGFβ-ZEB1/ZEB2 axis. As a transcriptional target of TGF-β, lncRNA-ATB competes with the miR-200 family, which eliminates EMT, resulting in up-regulation of ZEB1/ZEB2 expression. Our results are similar to those in other studies. LncRNA ZFAS1 was amplified at the genomic DNA level and competitively bound to miR-150, inhibiting ZEB1, MMP14 and MMP16 gene expression (87). STAT3-activated lncRNA HOXD-AS1 inhibited SOX4 mRNA expression by competing with miR-130a-3p to promote HCC lymph node metastasis (88).

## EMT-mediated chemotherapeutic resistance

**Figure 3:**
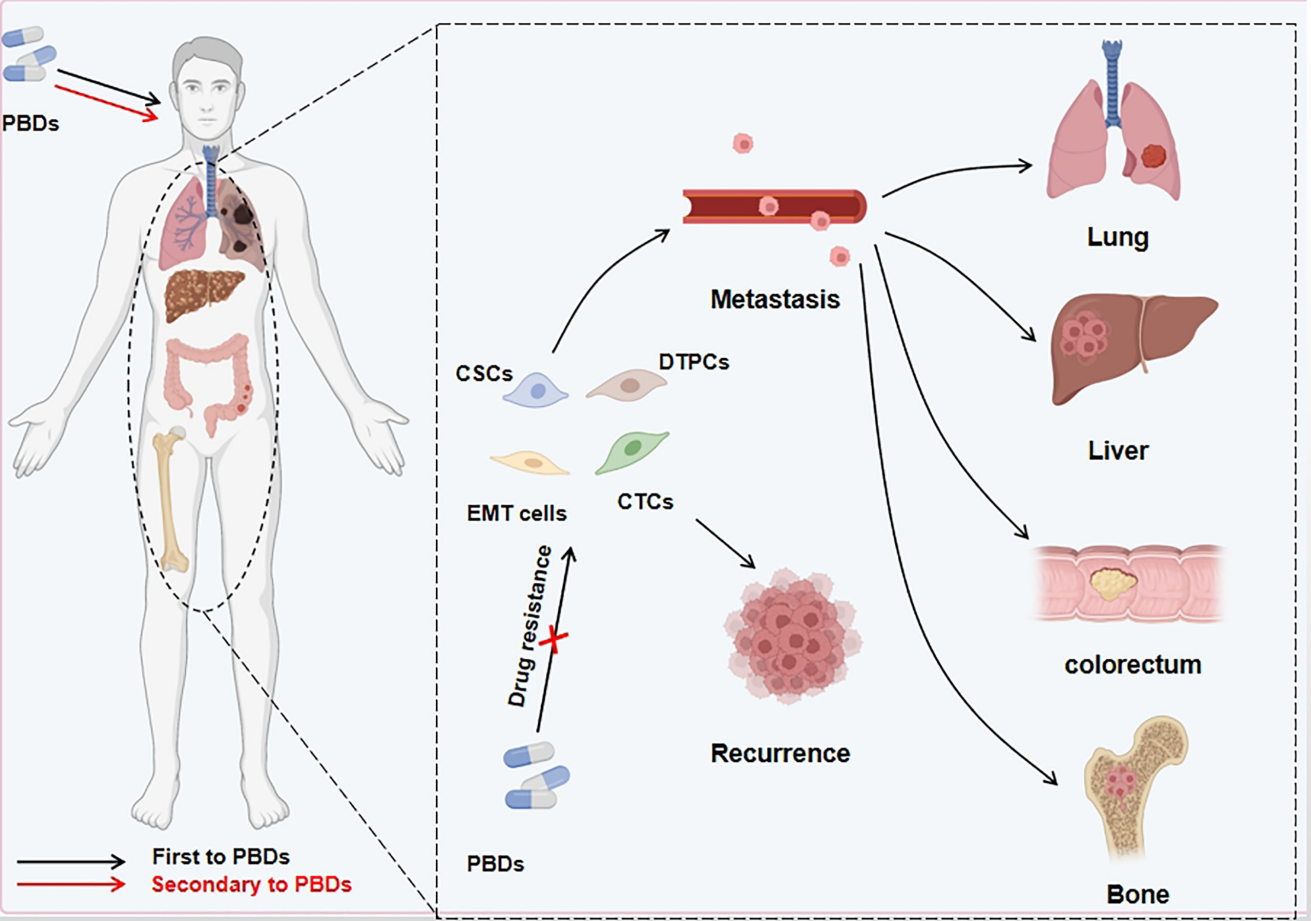
PBDs induce drug resistance through EMT in cancer cells. Platinum-based drugs can induce EMT through multiple sites/pathways and are either fully or partially transformed. In addition to the mesenchymal-like phenotype of tumor cells, a small number of other phenotypes appear during this process. Other cell types include persistent drug-resistant cells, cancer stem cells, and circulating tumor cells. These EMT-related cells can evade the deadly effect of PBDs, migrate to distant tissues and organs *via* the blood circulation and other routes, and cause *in situ* tumor relapse and metastatic foci after stopping medication.

EMT is characterized by the loss of tightly knit epithelial cells and transdifferentiation into mesenchymal cells. In recent years, meta-analysis of existing research data about the connection between gene expression and curative cancer effects showed that EMT-related genes were closely linked to resistance to certain kinds of treatment. For instance, an analysis of data from the clinical samples of breast cancer patients demonstrated that resistance to chemotherapy was an important cause of gene regulation of stromal cells, and the expression of these genes was mediated by EMT program activation (89). In addition, seventy-six EMT marker genes were used to explore gene expression and the effect of PI3K inhibitors in non-small cell lung carcinoma (NSCLC), and to predict resistance to these agents and assess prognosis (90). Gene changes can induce the generation of specific cell types as well as changes in phenotype, EMT-related cells, such as partial EMT cells (named hybrid EMT cells), drug-tolerant persister cells, and CSCs, which have a crucial function in creating drug resistance (**Table 3**). Next, we will look at how these cells are related to EMT and how to deal with PBD resistance (**Figure 3**).

**Table 3:** Phenotypic differences of CSCs, DTP cells and pEMT cells.

Type of cell	Markers	Type of cancer	Ref
CSCs	CD44 and CD133	Breast cancer	(91)
	CD44	Gastric cancer	(92)
	CD51	Prostate cancer	(93)
	CD133	Lung cancer	(94)
	CD133, nestin, and A2B5	Glioblastoma	(95)
	CD34	AML	(96)
DTP cells	ABCB5	Melanoma	(97)
	CD133		(98)
	CD133	Gastric cancer	(99)
	CD271	Osteosarcoma	(100)
	ALDHs	Brain cancer	(101)
pEMT cells	L1CAM	Colorectal cancer	(102)
	CD106, CD61 and CD51	Squamous cell carcinoma	(103)
	S100A6	Breast cancer	(104)
	NRF2	Non-small-cell lung carcinoma, Bladder cancer	(105)
	VEGF	Esophageal squamous cell carcinoma	(106)
	Cathepsin B	Salivary adenoid cystic carcinoma	(107)

### Partial EMT cells

Under normal physiological conditions, the EMT program is involved in embryonic development and wound repair, but tumor cells often enhance their invasive and metastatic capabilities by activating EMT, and especially to mount a resistance to chemotherapy drugs that ensures their survival (108). The EMT program involves the loss of junctional tightness between epithelial cells and their gradual differentiation into mesenchymal cells. Because mesenchymal cells have motility and are capable of degrading extracellular matrix (ECM) proteins, these cells can invade the surrounding tissues and metastasize to distal sites (109). Therefore, EMT is considered a sign that the tumor is progressing to a higher stage. Researchers have found that there is an intermediate state in the EMT process, in which cells have both epithelial and mesenchymal phenotypes. These are called partial EMT cells, and experimental and clinical results have shown that such cells are more malignant and drug-resistant than completely differentiated EMT cells (110).

Analysis of clinical data showed that most cancer rarely undergoes adequate transformation (3, 111). Non-small-cell lung cancers (NSCLC) patients were usually treated with gefitinib and erlotinib, which are epidermal growth factor receptor (EGFR) and tyrosine kinase inhibitors (112). However, EGFR resistance was usually accompanied by a partial EMT phenotype with co-expression of vimentin and E-cadherin. Similar evidence emerged from another clinical trial, where the epithelial cell subset and the mesenchymal cell subset were more sensitive to cisplatin, paclitaxel, and salinomycin than partial EMT cells (high CD44, high CD24, low EpCAM) in oral squamous cell carcinoma (OSCC) (113). These results indicate that, to some extent, partial EMT cells may have a greater chance of developing drug resistance. To verify the correctness of this view that partial EMT cells are more likely to develop drug resistance than completed EMT cells, we selected docetaxel-resistant PC-3 with complete EMT and DU145 with partial EMT cell lines for comparative experiments. PC-3 appeared to undergo apoptotic death and DU145 was resistant to treatment with salinomycin (114). Similarly, this hypothesis was validated by clinical results in which cancer cells altered their metabolic program to meet changing nutrition and energy requirements. This metabolic change began when tumor cells started the EMT program and was most robust in the partial EMT state. Autophagy is divided into protective autophagy and lethal autophagy, and the condition is enhanced in the partial EMT state. During chemotherapy or the loss of nutrients or growth factors, the protective autophagy activity in tumor cells was enhanced by AMPK expression to ensure lysosomal function (115, 116). This conclusion was confirmed in clinical studies as well as by *in vitro* research. For example, inhibiting protective autophagy promoted the release of arginase 1 (Arg1) from the liver, which promoted arginine synthesis and stimulated cancer cell growth (117). In clinical practice, enhanced autophagy promoted the occurrence and development of autochthonous pancreatic cancer (118). In addition, other experiments have shown that the presence of an epithelial cell adhesion molecule (EpCAM) on the surface of epithelial cells could induce the production of multidrug-resistant proteins (MDRPs) (119); however, cells that have completed the EMT lose this phenotype, leading to a reduction in the production of MDRPs. Although we know that partial EMT plays an important role in tumor resistance, an interesting phenomenon provides us with new ideas about the function of partial EMT. In the whole EMT lineage, partial EMT cells located at intermediate sites showed greater plasticity than fully transitioned EMT cells (120). We hypothesized that different degrees of EMT and different physical and chemical properties caused by differences in phenotype and morphological structure had different degrees of influence on tumor invasiveness, metastasis, and drug resistance.

### Cancer stem cells

CSCs have been considered an important factor in the sensitivity and tolerance to chemotherapy. Clinical practice demonstrated that CSCs existed in various tumor tissues, and the proportion of CSCs was significantly higher than that of the tumor before chemotherapy (121). However, the mechanism of how CSCs are produced is still unclear. With the introduction of the EMT, it is easy to determine whether CSCs and EMT are always found together in pathological tissue and to study the interaction between EMT and CSCs, because EMT induces and maintains CSCs and CSC phenotypes.

One of the possible explanations for the secreted proteins of carcinoma cells is that EMT leads to autocrine signaling loops. Multiple studies have revealed that autocrine signaling loops had the necessary activity to induce and maintain the characteristics of stem cells. EMT activation promoted the work of autocrine signaling loops to maintain the stem cell phenotype *via* the TGF-Smad and Wnt-catenin pathways in the secretome, which was secreted by transformed HMLER human mammary epithelial cells (122). Non-CSCs also had the plasticity necessary to promote cancer cell dedifferentiation, and this process depended on the occurrence of EMT (123). For example, some secreted extracellular vesicles and macromolecules such as microRNAs and cytokines, were released by EMT to induce differentiated tumor cells to dedifferentiate into CSCs (124). Intracellular signaling pathways and cytokines participated in the EMT process to generate the CSC phenotype. The tumor-suppressor protein, p53, is restrained by the expression of Snai1, which is linked to the tumorigenic ability of cancer cells *via* the Snai1-histone deacetylase 1 (HDAC1)-p53 complex. However, this ability could be uncovered by the downregulation of Tp53, which encoded p53 (125). EMT was first discovered during embryonic development and required TGF-β pathway activation (126). Researchers also found that TGF-β, IL-6, and hepatocyte growth factor (HGF) could induce the EMT process and enhance the conversion of the differentiated cell phenotype to CSC characteristics (127, 128). In addition, it has been found that excessive EMT activation led to maximal tumor-initiating ability and chemotherapy resistance (129). A partial EMT program may contribute to the frequent and substantial production of CSCs. In a mouse model of breast cancer, CSCs had different phenotypes, called epithelial-like CSCs and mesenchymal-like CSCs. These cells had a greater ability to seed cancer cells to distant tissues (130, 131). Moreover, the combination of epithelial and mesenchymal phenotypes of CSCs enhanced the progression of tumor drug resistance and metastasis (132). Although the relationship between EMT and CSCs provided a partial explanation, the hypothesis that the partial EMT could acquire more stem phenotypes than the complete EMT, and induce CSCs that were related to specific cell types and epigenetic abnormalities deserves further exploration.

CSCs have some physical and biochemical properties that influence sensitivity to clinical treatments, including chemotherapy, radiotherapy, molecular targeting, and immunotherapy. The first important feature is the ability to self-update, which optimizes tumorigenesis and differentiation in multiple types of cells, including drug-resistant cells. Recent research discovered that many signaling pathways, such as Wnt and Notch, engaged in self-renewal activation. for example, in breast cancer (133). PBDs induced EMT *via* the Notch pathway, and RO4929097, a Notch1 inhibitor, could repress N-cadherin and CD44 to produce better patient outcomes (134). In the tumor microenvironment (TME) after Notch pathway activation, the CSCs were ‘self-updated’ compared to the non-EMT environment. Signaling through Notch and PI3K/AKT/mTOR stimulated quiescent CSCs to progress into the S-phase of the cell cycle (135, 136) and tumor tissue exhibited higher resistance to radiotherapy and chemotherapy in the presence of active CSCs. The body of preclinical and clinical observations shows that radio-chemotherapy effectively eliminated most non-CSC cells but did not appreciably reduce the number of CSCs (137–139), which was why CSCs could continue to enter the G0 phase and remain there for a long period. These dormant cancer cells were not affected by the drugs, and CSC resistance to targeted treatment and immunotherapy was significantly higher than that of non-CSCs (140).

### Drug-tolerant persister cells

Research has determined that the entry into drug-tolerant persister (DTP) states of cancer cells is an adaptive response to chemotherapy with PBDs (141). The emergence of this state is currently subject to several hypotheses, including that DTP cancer cells are already present in tumor tissue and are selected for proliferation after drug treatment (142). Another likely reason is that the production of DTP cancer cells is induced by activation of cell reprogramming rather than through survival of the fittest by drug selection (143). This transformation process has been widely observed in clinical research and is most commonly considered to be EMT. For example, cancer tissues had abundant DTP cancer cells when treatment activated EMT (144). Therefore, the close relationship between DTP cancer cells and EMT was an essential factor in the multidrug resistance of these cells to tumor therapy.

DTP cancer cells have some characteristic phenotypes that help them evade the killing effects of drugs, such as reversible biological capacity, slow cell cycling, and proliferative activity (143, 145, 146). For example, a slowly cycling cell population that expressed high levels of the histone H3K4 demethylase, JARID1B, was preserved after chemotherapy in melanoma (147). In addition, several researchers reported that cancer cells with DTPs exhibited a stem-like phenotype to reduce drug influence; thus, EMT programs contributed to inducing and maintaining the CSC phenotype. This DTP phenotype allowed cancer cells to acquire plasticity, pluripotency, self-renewal capacity, and transferability, and to enter the G0 phase or slow-cycling state, which protected them from cytotoxic agents (148, 149). In addition, the activation of the Wnt/β-catenin pathway was found in the DTP cancer cells (150). These results also indirectly proved that the emergence of DTPs was closely related to the EMT process. Osimertinib, an EGFR tyrosine kinase inhibitor, upregulated the AXL gene-inducing EMT program. AXL bound to its ligand (GAS6) and promoted formation of DTP cancer cells by increasing the expression of DNA repair proteins to reduce DNA damage (151, 152). Mesenchymal EGFR mutant cancers survived initial EGFR inhibitors because fibroblast growth factor receptor 1 (FGFR1) was expressed on the cell surface (153). MiR-99b was activated when tumor cells underwent EMT reprogramming, and as FGFR3 was a target of miR-99b, it participated in the induction of DTP cancer cells (153, 154). In addition to FGFR1 and FGFR3, studies have shown that insulin-like growth factor 1 (IGF-1) and the lipid hydroperoxidase, GPX4, could activate the PI3K/AKT and ERK pathways to induce the EMT program, and IGF-1R phosphorylation in cancer cells led to the production of DTPs (145). TGFβ2 linked the EMT program to fatty acid metabolism in an acidic environment (155). In the EMT process, the epithelial cells transformed into mesenchymal cells, gaining motility and losing cell-cell adhesion, making them more capable of entering the circulatory system. Recent research has shown that persistent subsets of proliferative cells in CTCs—cycling persister cells (CPCs)—not only survived cancer drugs but also maintained their ability to grow and multiply with continuing drug treatment. Moreover, single-cell RNA sequencing and metabolic analysis demonstrated that CPCs enhanced antioxidant gene capacity by increasing glutathione metabolism. It was also found that CPCs were dependent on fatty acid-based metabolism rather than glucose-based metabolism (156). Therefore, CTCs with EMT activation are more likely to transform into CPCs, resulting in drug resistance, distant recurrent lesions, and metastasis.

## Targeting the EMT for cancer therapy

**Figure 4:**
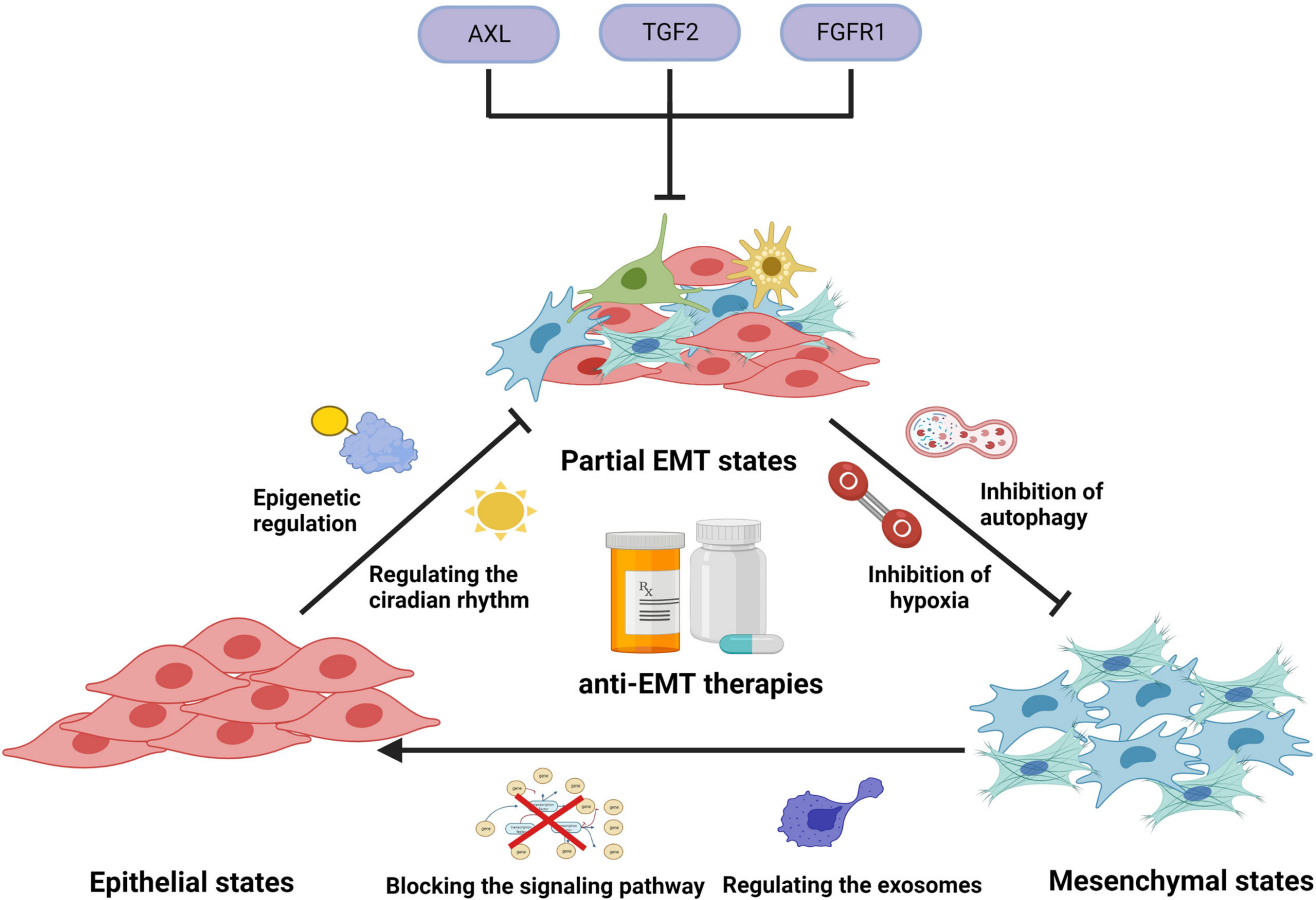
Illustration representing several anti-EMT therapies to overcome drug resistance. In clinical applications, platinum-based drugs can cause the induction and development of EMT by activating EMT-related signals, regulating circadian rhythms, regulating epigenetics, causing hypoxia, inducing autophagy, and stimulating exosome secretion, thereby generating tumor cells’ resistance to PBDs. Use of modality-related inhibitors can prevent and reverse EMT, enhancing the efficacy of PBDs. In addition, some small molecules can target EMT cells and prevent them from entering a drug-resistant state.

It has been proven that in the development of EMT, cancer cells generated new phenotypes, enabling them to survive PBD treatment. To eliminate drug resistance caused by EMT, different strategies to target the EMT program, such as preventing EMT initiation, targeting partial EMT cells, and reversing the EMT process, have been pursued (**Figure 4**).

### Preventing EMT initiation

Tumor cells develop EMT after chemotherapy due to alterations in signaling pathways, circadian rhythms, and epigenetic regulation, making the tumor cells more malignant and eventually drug-resistant (157). For this reason, blocking the induction of EMT in tumor cells is crucial. Chemotherapy results in abnormal activation or inhibition of signaling pathways in tumor cells, and these abnormal signaling pathways can lead to EMT. We have previously described in detail the signaling pathways involved in platinum-induced EMT, including Wnt, TGF-β, Notch, NF-kB, and hedgehog. Blocking any of the links in the signaling pathway of drug-induced EMT can affect the development of EMT in tumor cells to varying degrees. It was shown that the CXCL/CXCR2 axis was activated in resistant cells after cisplatin treatment, allowing CXCR2 to be highly expressed in human lung cancer tissues. Overexpression of CXCR2 promoted EMT by activating the p38/ERK MAPK pathway. SB225002, a selective CXCR2 inhibitor, blocked CXCR2 expression, thus preventing EMT formation (51). In addition, hematopoietic PBX interacting protein (HPIP) was overexpressed in high-grade primary ovarian tumors, which increased Snail stability by activating the PI3K/AKT pathway and inhibiting the expression of E-cadherin by phosphorylating glycogen synthase kinase-3β (GSK-3β), thereby inducing EMT. Therefore, it is reasonable to attempt to inhibit the formation of EMT by knocking down HPIP using PI3K and AKT inhibitors (158). Reducing the expression of Snail could also have an inhibitory effect on the formation of EMT (159).

Epigenetic regulation plays an important role in cancer progression. Ten-eleven translocation 1 (TET1), an important DNA demethylase, was found to be overexpressed in cisplatin-resistant ovarian cancer cells and could induce partial EMT by increasing vimentin expression through demethylation of the vimentin promoter. Thus, we could inhibit EMT formation by downregulating TET1 expression using TET1 siRNA (160). Another study found that cisplatin-resistant ovarian cancer cells had increased expression of histone deacetylase (HDAC), which resulted in histone hypoacetylation and suppression of many genes, leading to EMT induction and subsequent PBD resistance. Consequently, the HDAC inhibitor sodium butyrate (Nabu) could be used to restore histone acetylation, allowing tumor cells to upregulate E-cadherin expression and thus inhibit EMT formation. In addition, the combination of Nabu and cisplatin enhanced the toxicity of cisplatin on tumor cells (161).

Hypoxic conditions can occur because of rapid tumor growth and lack of blood vessel formation. Hypoxia drives adaptive changes that can lead to malignant tumor transformation. In NSCLC, hypoxia significantly upregulated antizyme inhibitor 2 (AZIN2) expression by increasing the binding of HIF-1α and AZIN2 promoters, resulting in decreased E-cadherin expression and increased N-cadherin and vimentin expression, thereby promoting EMT (162). Therefore, maintaining oxygenation at the appropriate level could be a viable strategy for preventing EMT initiation in tumor cells. In treating cisplatin-resistant gastric cancer with Danggui-Sayuk-Ga-Osuyu-Saenggang-tang (DSGOST), an accumulation of GFP-LC3 puncta was induced, which promoted the release of exosomes through activation of autophagy, thereby inducing EMT. Consequently, the activation of DSGOST-mediated EMT markers, including N-cadherin, Snail, Slug, vimentin, β-catenin, p-Smad2, and p-Smad3, could be blocked by inhibiting autophagy, which in turn inhibited EMT formation (163).

Traditional Chinese medicine (TCM) research on the EMT process is only in the initial stages, but we can still identify new potential drugs and strategies for inhibiting the formation of EMT from existing TCM extracts and monomers. As a prime example of the value of TCM, curcumin often appears in various prescriptions. In recent years, studies have shown that curcumin inhibited TGF-β-induced EMT through the PPARγ pathway. Other researchers found that curcumin could effectively inhibit the NF-κB pathway to reduce the expression of EMT-related genes (164–166). In addition, ginsenoside Rg3, triptolide, and resveratrol had similar effects (167, 168). Therefore, analyzing the TCM extracts for active compounds is a worthy effort that could lead to new drugs for translation into clinical treatments.

### Targeting partial EMT cells.

The intermediate state on the way to complete EMT is called partial EMT, the essential feature of which is the simultaneous expression of E-cadherin and vimentin. Multiple studies have shown that cancer cells with coexpression of E-cadherin and vimentin led to poorer prognosis compared with those that expressed E-cadherin or vimentin alone, or neither (169). Therefore, when the tumor tissue presents a partial EMT state and is highly resistant to multiple drugs, the elimination of some or all of them could increase the therapeutic effect and prolong the survival of patients to a certain extent. In addition to these EMT markers, a number of other markers were also found associated with partial EMT, such as integrin beta 4 (ITGB4), interrogating the grainyhead-like 2 (GRHL2), ferroptosis suppressor protein 1 (FSP1), and ZEB1. These markers were also associated with shorter overall survival, poor relapse-free and disease-free survival (132, 170, 171). Whole-genome CRISPR screening revealed that mesenchymal EGFR mutant non-small cell lung cancers highly express FGFR1, which promotes DTPs. Combining EGFR and FGFR inhibitors could block the development of persistent resistance and drug-tolerant cell survival (153). Some molecules, such as recombinant AXL receptor tyrosine kinase (AXL), TGF2, and FGFR1, could be targeted to inhibit cells in EMT states from becoming DTPs, which could be a novel approach for correcting EMT-induced drug resistance. In addition, the cancer cells of the EMT process released proinflammatory cytokines, inducing drug resistance. For example, comprised of chemokine ligand 21 (CCL21) stimulated the AKT/GSK3β/Snail pathway, which promoted the expression of multidrug resistance proteins such as P-glycoprotein1 (172). Therefore, we speculated that preventing partial EMT cells from secreting small molecules might inhibit the production of drug-resistant proteins. In addition, it is also interesting to see whether the protective molecules secreted by these cells play a role in immune cell aggregation to engulf chemotherapeutic drugs and prevent immune escape.

### Reversing the EMT process.

The EMT program is positively correlated with poor cancer prognosis. Therefore, taking the reversal of EMT as the starting point for resistance to cancer should increase the success rate of chemotherapy. For example, the ionophore antibiotic salinomycin upregulates the E-cadherin gene while reducing the expression of vimentin in CD133+ colorectal cancer cells and restoring cancer cell sensitivity to chemotherapy. This research also found that CD133 was a target of salinomycin (173). Therefore, combining salinomycin and PBDs could reverse the drug-induced EMT process and specifically target CD133+ CSCs. The specific signaling pathways play a role in both the induction of EMT and the drug resistance induced by EMT; thus, turning off the activated pathways should reverse EMT and eliminate drug resistance (174). A combination of cisplatin and BEZ235, an ATP-competitive dual inhibitor of PI3K and mTOR, could reverse the EMT program, induce apoptosis, and decrease the number of resistant cells (175).

Mesenchymal stem cells secrete exosomes that increase the mRNA levels of CK-19 and E-cadherin and decrease the expression of vimentin. In addition, exosomes reduce phosphorylation of the upregulated proteins, vimentin, TGF-β1, and Smad2 (176). Further studies suggest that miRNAs play an essential role in this process. For example, exosomes derived from mesenchymal cells can transfer miR-182-5p and miR-23a-3p into EMT cells and target Ikbkb and ubiquitin specific peptidase 5 (Usp5) to repress IKKβ ubiquitination, leading to the inhibition of NF-κB signaling and the reversal of EMT (177). The secretion by mesenchymal cells of specific molecules that reverse the EMT process could partly explain the presence of partial EMT. It might be possible to enhance the secretion of these substances and the formation of exosomes to eliminate tumor tissue resistance to chemotherapy.

## Conclusions and perspectives

Research in the field of EMT has evolved from promoting normal development and wound repair to having a significant and complex impact on tumor resistance. On the one hand, EMT can induce the generation of cells with different phenotypes, which are resistant to PDBs to different degrees. On the other hand, the EMT process can induce tumor cells to produce multidrug-resistant proteins to evade the killing effect of drugs. It has been recognized that the induction of EMT required the activation of multiple signaling pathways, offering several opportunities for therapeutic intervention to inhibit the occurrence and development of the EMT process, and even to reverse it. However, the combination of EMT inhibitors and PBDs has not achieved the desired effect in clinical practice. This may be related to the synergistic effect of multiple signaling pathways, the constantly changing tumor microenvironment, or stress-induced metabolic changes in the tumor. New research findings together with advances in technology could better promote the development of new drugs so that PBDs combined with radiotherapy could be precisely targeted to ensure a more substantial inhibitory effect on the EMT process with fewer toxic side effects. Moreover, the application of new technology and bioinformatics methods has led to a deeper understanding of EMT, with the discovery of new proteins and new pathways, checkpoints and bypasses, which play important roles in resistance to chemotherapeutic drugs. Current research on EMT has been focused on identifying and neutralizing the EMT-related proteins involved in drug resistance. Highlights of the recent research include the effects of CSCs, CTCs, and DTPs on tumor recurrence, metastasis, and drug resistance, which have promoted an in-depth exploration of the relationship between these cells’ metabolism and EMT. Another fruitful area of research has been the effects of the extracts and individual components of traditional Chinese medicines on EMT. The results of these studies are expected to be the keys to breaking the bottleneck of EMT-induced tumor resistance. In the coming decades, research on EMT and the mechanism of drug resistance will deepen our understanding of how to solve the resistance to PBDs and reveal ways to Kibbe make these agents safer and more effective in clinical practice.

## Author contributions

XD and ML wrote the manuscript. JL made the figures. ZS and KX revised the manuscript. All authors contributed to the article and approved the submitted version.

## Funding

This work was supported by grants from Beijing Xisike Clinical Oncology Research Foundation (2020HX037/Y-MSD2020-0354).

## Conflict of interest

The authors declare that the research was conducted in the absence of any commercial or financial relationships that could be construed as a potential conflict of interest.

## Publisher’s note

All claims expressed in this article are solely those of the authors and do not necessarily represent those of their affiliated organizations, or those of the publisher, the editors and the reviewers. Any product that may be evaluated in this article, or claim that may be made by its manufacturer, is not guaranteed or endorsed by the publisher.
